# Elucidating the Influence of Tumor Presence on the Polymersome Circulation Time in Mice

**DOI:** 10.3390/pharmaceutics11050241

**Published:** 2019-05-20

**Authors:** Robin M. de Kruijff, René Raavé, Annemarie Kip, Janneke Molkenboer-Kuenen, Stefan J. Roobol, Jeroen Essers, Sandra Heskamp, Antonia G. Denkova

**Affiliations:** 1 Radiation Science and Technology, Delft University of Technology, 2629 JB Delft, The Netherlands; A.G.Denkova@tudelft.nl; 2 Radiology and Nuclear Medicine, Radboud University Medical Center, 6525 GA Nijmegen, The Netherlands; rene.raave@radboudumc.nl (R.R.); Annemarie.Kip@radboudumc.nl (A.K.); Janneke.Molkenboer-Kuenen@radboudumc.nl (J.M.-K.); Sandra.Heskamp@radboudumc.nl (S.H.); 3 Molecular Genetics, Oncode Institute, Erasmus University Medical Center, 3000 CA Rotterdam, The Netherlands; s.roobol@erasmusmc.nl (S.J.R.); j.essers@erasmusmc.nl (J.E.); 4 Radiology and Nuclear Medicine, Erasmus University Medical Center, 3000 CA Rotterdam, The Netherlands

**Keywords:** radiolabeled polymersomes, clodronate liposomes, circulation time, healthy and tumor-bearing mice, macrophages

## Abstract

The use of nanoparticles as tumor-targeting agents is steadily increasing, and the influence of nanoparticle characteristics such as size and stealthiness have been established for a large number of nanocarrier systems. However, not much is known about the impact of tumor presence on nanocarrier circulation times. This paper reports on the influence of tumor presence on the in vivo circulation time and biodistribution of polybutadiene-polyethylene oxide (PBd-PEO) polymersomes. For this purpose, polymersomes were loaded with the gamma-emitter ^111^In and administered intravenously, followed by timed ex vivo biodistribution. A large reduction in circulation time was observed for tumor-bearing mice, with a circulation half-life of merely 5 min (*R*^2^ = 0.98) vs 117 min (*R*^2^ = 0.95) in healthy mice. To determine whether the rapid polymersome clearance observed in tumor-bearing mice was mediated by macrophages, chlodronate liposomes were administered to both healthy and tumor-bearing mice prior to the intravenous injection of radiolabeled polymersomes to deplete their macrophages. Pretreatment with chlodronate liposomes depleted macrophages in the spleen and liver and restored the circulation time of the polymersomes with no significant difference in circulation time between healthy mice and tumor-bearing mice pretreated with clodronate liposomes (15.2 ± 1.2% ID/g and 13.6 ± 2.7% ID/g, respectively, at 4 h p.i. with *p* = 0.3). This indicates that activation of macrophages due to tumor presence indeed affected polymersome clearance rate. Thus, next to particle design, the presence of a tumor can also greatly impact circulation times and should be taken into account when designing studies to evaluate the distribution of polymersomes.

## 1. Introduction

The use of nanoparticles in medicine is increasingly gaining attention, mainly in cancer-related therapies, but also extending to HIV, asthma, and other infections [1]. While only a few nanoparticle-based drugs have made the translation to the clinic [2], they show great promise in, for example, reducing toxicity [3,4]. They provide a versatile platform for theranostic approaches, allowing for easy surface modification and/or encapsulation of drugs and contrast agents. There are a number of well-established factors which have to be taken into account when designing nanocarrier studies resulting in the desired tumor accumulation of the nanocarrier in question. Many nanocarrier designs rely on passive tumor targeting, making use of the leaky tumor vasculature combined with poor lymphatic drainage (the enhanced permeability and retention (EPR) effect). The accumulation of nanoparticles through passive targeting therefore depends on the physiological properties of the tumor [5], as the EPR effect may differ between tumor models [6]. Tumor models which exhibit high EPR effect include CFPAC-1 [7], MDA-MB-231 [8,9], and 4T1 [10,11]. However, for sufficient accumulation at the tumor site through passive targeting, it is also essential to design long-circulating nanocarriers, as one of the main challenges for targeted drug delivery is their fast uptake by the mononuclear phagocyte system (MPS). Factors involved with the uptake of nanoparticles by the MPS are degree of PEGylation (polyethylene glycol), size, and surface charge [12,13]. PEGylation has been shown to minimize opsonization for a number of nanoparticles, including liposomes and polymer-based nanoparticles [14], resulting in a 1–2 order of magnitude increase in circulation time [15,16,17]. The optimal vesicle size can depend on the nanocarrier type; for example, whereas the optimal liposome diameter appears to be between 50 and 200 nm [18,19], polymersomes have a much stricter size constraint, with optimal circulation times around 90 nm while 120 nm vesicles are quickly cleared from the blood [20]. While many studies have focused on the influence of nanoparticle properties as well as tumor models, the influence of tumor presence on nanoparticle circulation time has barely been researched. This is of utmost importance in selecting preclinical models used for investigating the circulation time and tumor-targeting properties as these characteristics may strongly be affected by presence of tumor cells [21,22].

Previously, we have studied the use of polymersomes for the application of radionuclide therapy. Polymersomes [21,23,24,25] are versatile nanocarriers which allow easy modification of physicochemical properties creating a large number of possible applications [26]. They are often compared to liposomes, which are composed of phospholipids instead of block copolymers, due to their similar amphiphilic nature [27]. Their circulation half-life has in fact been found to be similar to that of stealth liposomes [28]. However, their ability to efficiently encapsulate and retain radionuclides in their aqueous compartment, including their potential application in alpha radionuclide therapy where they have proven to retain daughter nuclides better than their lipid counterparts [23,24], makes them an interesting nanocarrier model for targeted alpha therapy. Many in vivo studies with polymersomes have been performed in BALB/c or NCr nude mice, which have a severely comprised immune system. However, in an earlier study, we have observed reduced polymersome circulation time upon the presence of a tumor [21]. Despite the fact that these mice were immunocompromised, they do contain subsets of immune cells such as macrophages, which are known to be mainly responsible for nanoparticle removal from circulation. The presence of a human tumor may be able to activate these macrophages, which in turn may influence the circulation time of polymersomes. In this paper, we have therefore studied the influence of tumor presence on the circulation time of polymersomes labeled with the gamma-emitting radionuclide ^111^In through periodic blood sampling. Furthermore, the effect of the MPS on the circulation time was examined through the administration of clodronate liposomes which are known to cause macrophage depletion [29].

## 2. Materials and Methods

### 2.1. Chemicals

PBd-PEO (polybutadiene-polyethylene oxide) block copolymer with a *M*_w_ of 1900–900 g/mol was purchased from Polymer Source (Dorval, QC, Canada). ^111^In was obtained from Covidien (Petten, The Netherlands). The chlodronate liposomes were purchased from Liposoma B.V. (Amsterdam, The Netherlands). The PD10 size exclusion columns were obtained from GE Healthcare (Hoevelaken, The Netherlands). All other chemicals were purchased at Sigma Aldrich (Zwijndrecht, The Netherlands).

### 2.2. Polymersome Preparation

Polymersomes with an average diameter of 80 nm were prepared by the solvent displacement method. Here, 1 mL PBS (phosphate-buffered saline) buffer solution containing 1 mM DTPA at pH 7.4 was added to a 20 mg/mL block copolymer solution in 1 mL acetone using a syringe pump under magnetic stirring at 300 rpm. Subsequently, the acetone was evaporated using a rotavapor (Büchi, Switzerland) under reduced pressure (100 mbar) for 15 min, and 1 mL PBS was added to bring the final concentration to 10 mg/mL block copolymer. Before radiolabeling, remaining free DTPA was removed from the solution by passing it through a 30 × 1 cm (length (*L*) × diameter (*D*)) Sephadex G 25 medium mesh size extrusion column equilibrated in PBS at a pH of 7.4.

### 2.3. Radiolabeling and Radionuclide Retention

Polymersomes were radiolabeled with ^111^In, where 20 µL tropolone was added to approximately 200 MBq of ^111^InCl_3_ in pH 2 HCl and 200 µL 10 mM HEPES. After an incubation time of 10 min, 1 mL polymersome solution, from which free DTPA was removed, was added and incubated for 30 min. Subsequently, the unencapsulated ^111^In was removed by passing the solution through a PD10 column, where 0.5 mL fractions were collected and those containing the polymersomes were used for in vivo experiments.

To assess ^111^In retention in the vesicles, 500 µL of the ^111^In-containing polymersomes were incubated with either 500 µL 1 mM DTPA or 500 µL BALB/c mouse serum for 24 h at 37 °C. The polymersomes were separated from any free ^111^In-DTPA by passing them through a PD10 column, or separated from the serum by passing them through a Sepharose 4B column (31 × 1 cm (*L* × *D*)) and collecting 1 mL fractions.

### 2.4. Cryogenic Transmission Electron Microscopy (Cryo-TEM) and Dynamic Light Scattering (DLS)

Cryo-TEM images were obtained at an acceleration voltage of 120 keV using a Jeol JEM 1400 TEM (JEOL Ltd., Tokyo, Japan). The samples were prepared as follows: 4 μL 10 mg/mL polymersome solution was deposited on a holey carbon film (Quantifoil 1.2/1.3, Cu 200 mesh grids) supported on a TEM grid and blotted for four seconds. It was subsequently vitrified though rapid immersion in liquid ethane (Leica EM GP version 16222032, Leica Microsystems B.V., Amsterdam, The Netherlands), and then inserted into a cryo-holder (Gatan model 626, Gatan, Pleasanton, CA, USA).

DLS spectra were obtained by diluting the polymersome solution in PBS to a concentration of 0.01 mg/mL, after which it was placed in a toluene-filled, temperature-regulated bath (20 °C) in a DLS apparatus. The DLS consisted of a JDS Uniphase 633 nm 35 mW laser, a fiber detector, an ALV sp 125 s/w 93 goniometer, and a Perkin Elmer photon counter (Perkin Elmer, Groningen, The Netherlands), with an ALV-5000/epp correlator and software. The intensity autocorrelation function was determined at 90°, and the data was fitted using the Contin method [25].

### 2.5. Cellular Uptake Experiments

Fluorescent polymersome labeling was done using a fluorescent moiety attached to a lipophilic tail with optimal excitation at 551 nm and emission at 567 nm (PKH26, Sigma-Aldrich) according to manufacturer protocol. In short, 20 μL of polymersome solution (10 mg/mL) and 5 μL PKH26 dye (working concentration of 2.5E-5 M) were diluted in provided Diluent C to 100 μL end volume and incubated for 10 min. Excess PKH26 dye was removed using an Exosome Spin Column (Sigma-Aldrich) according to manufacturer protocol. After solidifying and removing excess PBS, 100 μL of the labeled polymersome solution was added to the column and centrifuged for 2 min at 750× *g*, leaving only labeled polymersomes in Diluent C solution. The stock solution was diluted to 1 mL for appropriate concentrations.

J774A.1 (mouse BALB/c monocyte macrophage) and MDA-MB-231 (human breast cancer) cells were cultured in RPMI 1640 supplemented 1% penicillin/streptomycin and 5 or 10% fetal calf serum. All cells were incubated at 37 °C in a water-saturated atmosphere with 5% CO_2_. For uptake experiments, cells were seeded on round coverslips of 18 mm in diameter and grown overnight. The cells were incubated with 30 μL of labeled polymersome solution, diluted in 1 mL of culture media (6 μg/mL of labeled polymersomes). Cells were washed with PBS and fixed with 4% para-formaldehyde for 30 min at 30, 60, and 120 min after polymersome addition. Fixed cells were mounted on tissue slides using Vectashield (VECTOR laboratories, Amsterdam, The Netherlands) with DAPI (4′,6-diamidino-2-phenylindole) for nucleus staining.

Confocal microscopy (SP5, Leica, Amsterdam, The Netherlands) was used to capture at least 150 cells per time-point for both cell lines. With the use of FIJI software, the number of nuclei (threshold and analyze particles function) and polymersomes (find maxima function) were counted in each image. Dividing polymersome counts by nuclei counts, the concentration of polymersomes per nucleus was determined.

### 2.6. Animals

The Dutch central committee on animal research and the local ethical committee on animal research of the Radboud University approved this study under protocol 2015-0071 (2 September 2015). All animal experiments were performed according to the institutional guidelines. Upon arrival, 6–8-week-old female BALB/cAnNRj-Foxn1^nu^/Foxn1^nu^ mice (Janvier Labs, Le Geneset-Saint-Isle, France) were randomly tattooed for identification and were acclimatized for ≥4 days before any experimental procedure. Mice had unlimited access to food and water and were maintained with 5–6 mice per cage in a controlled environment (22 ± 1 °C, 55 ± 10% humidity, 12 h dark/light cycle). Cages were weekly replaced by clean cages. Tumor-bearing mice were injected with 5 × 10^6^ MDA-MB-231 breast cancer cells in Matrigel, a model known for its leaky vasculature enabling the EPR effect [9], and tumors were grown for three weeks. Just before the intravenous injections, the tumor length, width, and height were measured with a caliper for tumor-bearing mice and tumor volume was calculated. Tumor size was used to block-randomize the mice over the different groups.

### 2.7. Blood Clearance

To accurately quantify the circulation time of polymersomes in healthy and tumor-bearing mice, two groups of mice (MDA-MB-231 tumor-bearing and non-tumor-bearing) were intravenously injected with 200 µL, 1 MBq ^111^In radiolabeled 3 mg/mL polymersomes in PBS. Small blood samples were taken of three randomly selected mice per group to measure the polymersome concentration at 1 min, 15 min, 30 min, 1 h, and 2 h postinjection. Four hours after injection, all animals were euthanized, and organs of interest were collected and weighed. The radioactivity was counted in an Automated Wizard Gamma Counter (Perkin Elmer). Three standard solutions of the ^111^In-polymersome solution were measured to allow determination of the injected dose per gram of tissue (%ID/g). Paired two-sided Student’s *t*-tests were used to compare the difference in organ uptake in healthy and tumor-bearing mice.

### 2.8. Clodronate Liposomes

To determine the effect of macrophages on polymersome circulation time, mice were injected with clodronate liposomes to deplete all macrophages. Four different groups were defined, and two groups (one with MDA-MB-231 tumors and one without tumors) were intravenously injected with 300 μL clodronate liposomes at 5 days and 24 h before the polymersome injection. The other two groups (one with MDA-MB-231 tumors and one without tumors) received the polymersomes only. After injection of the radiolabeled polymersomes (200 µL, 3 mg/mL polymersomes labeled with 1 MBq ^111^In), a small blood sample was collected at 30 min postinjection to measure activity of polymersomes in blood. Four hours after injection, the animals were sacrificed followed by ex vivo biodistribution as described previously. Tumors and parts of the liver and spleen were stored in formalin, and parts of the liver and spleen were frozen in TissueTek (Sakura Finetek, Alphen aan den Rijn, The Netherlands) in a tray containing isopentane on dry ice for immunohistochemistry.

### 2.9. Immunohistochemistry

Spleen and livers were fixed overnight in 4% formalin in PBS and embedded in paraffin using routine procedures. Then, 5 µm sections were deparaffinised with xylene, incubated in 3% H_2_O_2_ in methanol for 20 min to block endogenous peroxidase, and subsequently rehydrated using a descending ethanol series. Sections were incubated in 10 mM citrate buffer (pH 6.0) at 37 °C for 2 h and subsequently incubated in 0.075% trypsin (Sigma-Aldrich) in PBS at 37 °C for 7 min. Nonspecific binding was blocked by incubating the sections in 10% normal rabbit serum in PBS for 20 min and subsequently the sections were incubated overnight at 4 °C with an anti F4/80 antibody 1:1000 in PBS, 2% NRbS (Rat anti ms, hu F4/80;BM8 eBioscience). After washing with PBS, the sections were incubated for 30 min at RT with 200 times diluted biotinylated rabbit-anti-rat (Vector laboratories, cat.nr. BA-4001). Finally, an avidin–biotin complex was applied to the sections for 30 min, and they were subsequently incubated with Bright DAB (3,3′-diaminobenzidine) for 8 min at RT. Nuclei were stained by incubating the sections for 5 s with hematoxylin and a subsequent wash with tap water for 10 min. Finally, the sections were dehydrated with, consecutively, water, 50%, 70%, and twice 100% ethanol, and twice with xylene, after which they were mounted with Permount, dried, and imaged.

## 3. Results and Discussion

### 3.1. Circulation Time in Healthy Versus Tumor-Bearing Mice

When employing nanoparticles like polymersomes for tumor theranostics, the EPR effect is frequently used. Essential for sufficient tumor uptake through this form of passive targeting are nanoparticle size (<200 nm) and long circulation times (~24 h) [30]. Previous research by our group using Ncr nude mice bearing a U87 MG glioblastoma tumor indicated that the PBd-PEO polymersomes circulate for a relatively short time [21]. However, the circulation time was not quantitatively determined. SPECT images suggested polymersomes were completely cleared from circulation in tumor-bearing mice, while they still circulated in healthy mice at 1 h p.i. Here, we quantitatively investigated the circulation half-life of these 80 nm diameter polymersomes and determined the effect of tumor presence on nanoparticle circulation.

**Figure 1:**
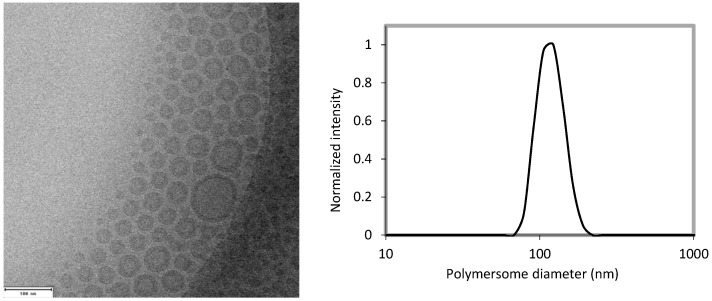
(**Left**): Characteristic Cryo-TEM (cryogenic transmission electron microscope) image of the 80 nm PBd-PEO (polybutadiene-polyethylene oxide) polymersomes; the scale bar represents 100 nm. (**Right**): A DLS (dynamic light scattering) measurement of these polymersomes.

**Figure 2:**
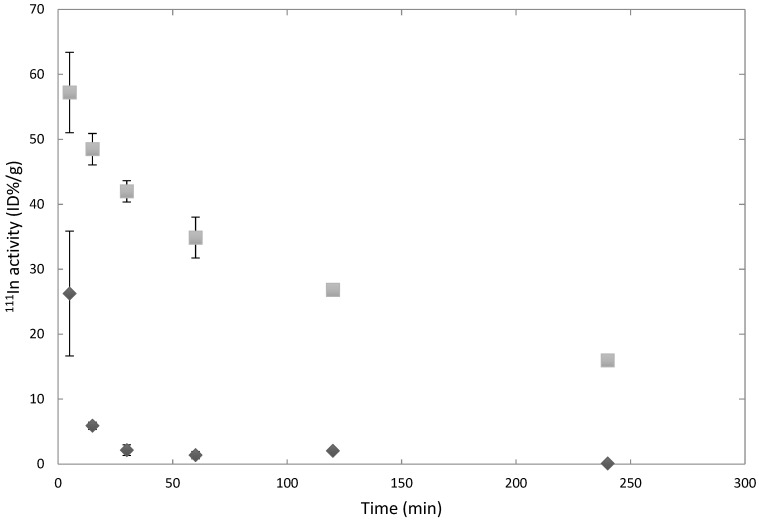
Circulation time of 80 nm polymersomes in BALB/c mice, either healthy (light grey) or bearing an MDA-MB-231 tumor (dark grey) over the course of 4 h (*n* = 3 mice per data point).

Figure 1 shows the characterization of the polymersomes used in these studies with DLS and Cryo-TEM. The radionuclide ^111^In was successfully encapsulated into the polymersomes with an efficiency of 94 ± 2%. The stability of the encapsulated ^111^In was determined by challenging the vesicles with either 1 mM DTPA at RT or mouse serum for 24 h at 37 °C. They displayed an ^111^In retention of 98.5 ± 0.9%. When incubated with BALB/c mouse serum, less than 1% of the ^111^In activity was associated with the serum fraction. Previous research by our group has established that the polymersomes themselves are not cytotoxic [31]. These polymersome formulations have thus proven stable enough to allow for further in vivo experiments. We were able to accurately determine circulation half-lives by taking blood samples at a number of time intervals as evident from Figure 2.

The activity levels have been decay-corrected and subsequently fitted with $A\left(t\right)=A_{0}e^{\frac{-t\ln2}{t_{1/2}}}$ to determine the circulation half-life. Here, *t*_1/2_ is the circulation half-life, *t* is the time in minutes, *A*(*t*) the activity in the blood at time *t*, and *A*_0_ the initial ^111^In activity. The circulation half-life has been fitted to be 117 min (*R*^2^ = 0.95) for healthy mice, and 5 min (*R*^2^ = 0.98) in tumor-bearing mice, which is a surprisingly large difference. For this experiment, the mice have been randomly placed in the different groups. Apart from the injection of the tumor cells in Matrigel in the tumor-bearing mice, the groups of mice were identical and received identical treatment, which indicates that the difference in circulation time stems from the tumor presence in the tumor-bearing mice. This dependence of the circulation time on tumor presence is not very well known as, to the best of our knowledge, only one other group has reported on this difference [22]. They observed an increase in M2-like macrophages in tumor-bearing mice as compared to healthy ones, and found that tumor presence drastically impacted the circulation time of their hydrogel nanoparticles while small molecules did not exhibit a difference in circulation time. Tumor presence has been shown to cause a shift in the normal immune system balance from Th1 to Th2 cytokines [32], which induce macrophages to differentiate into M-1 or M2-type macrophages, respectively [22]. Jones et al. found a remarkably large difference in nanoparticle clearance rate between the two macrophage types, where M2 macrophages result in much more rapid nanoparticle clearance [33].

**Figure 3:**
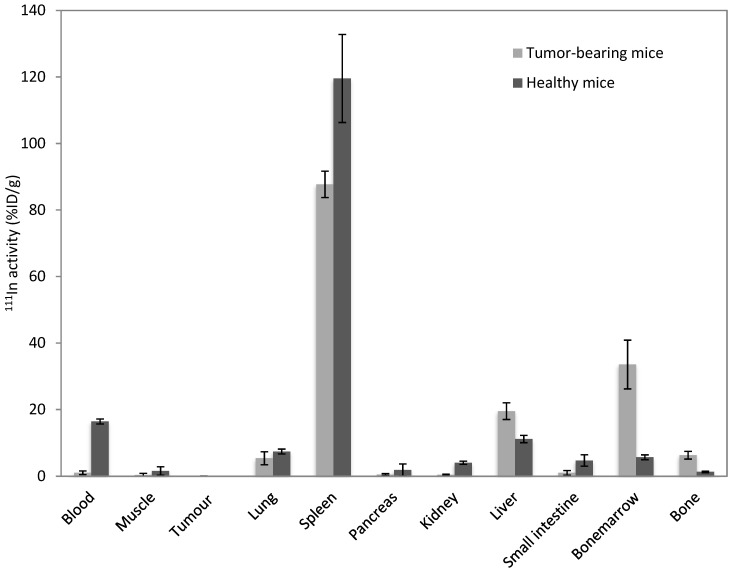
Biodistribution data of polymersomes with 80 nm diameter were injected intravenously in healthy female Balb/c nude mice (dark grey) or those bearing an MDA-MB-231 tumor (light grey). Polymersomes containing DTPA were labeled with 1 MBq ^111^In. The biodistribution was performed 4 h p.i.; bars represent mean with SD.

In Figure 3, the biodistribution data at 4 h p.i. is shown for 80 nm polymersomes in both healthy and tumor-bearing mice. Uptake in the spleen, liver, and bone marrow suggests that the main cause of this short circulation time has been uptake by the MPS, as macrophages are able to remove nanoparticles from the circulation within minutes [34]. Although nanoparticles typically accumulate in the spleen and the liver, depending on the surface characteristics, nanoparticles can be taken up by the mononuclear phagocyte cells in the bone marrow [35,36]. Significant differences in organ uptake (*p* < 0.005) can be observed in the blood, spleen, liver, and bone marrow.

Kai et al., who have studied the interaction of PEGylated PRINT (Particle Replication in Nonwetting Templates) hydrogel nanoparticles with immune cells in different organs, have shown an increase in bone marrow myelopoiesis in the presence of a tumor, and also found significantly higher liver uptake in tumor-bearing mice, though no large differences in uptake were found in the spleen. The difference in liver uptake was found to be associated mainly with a larger proportion of M2 macrophages, again due to the tumor presence [22]. These macrophages could also be responsible for the increase in liver uptake in the tumor-bearing mice in our study. There is, to the best of our knowledge, no indication in the literature why splenic uptake of nanoparticles should decrease upon the presence of a tumor. However, it could potentially be due to the enhanced rapid uptake of polymersomes in the liver, resulting in less time for the spleen to take up the polymersomes. This, in turn, can be linked to the significantly slower blood flow in the liver as compared to the systemic circulation, giving the liver macrophages, which are enhanced due to the tumor presence, relatively more time to accumulate nanoparticles [37]. Hence, we have further investigated the involvement of macrophages on the rapid clearance of nanoparticles.

### 3.2. Influence of Macrophages on Circulation Time

#### 3.2.1. Cell Experiments

**Figure 4:**
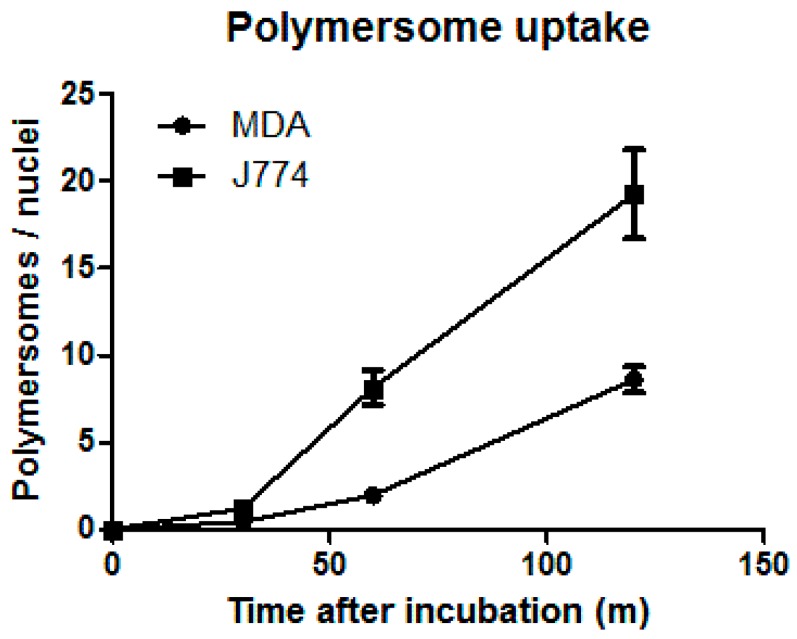
Uptake of polymersomes in a J774 macrophage cell line vs the MDA-MB-231 tumor cells. Cells were incubated with 6 µg of polymersome solution and were fixed at 30, 60, and 120 min postaddition of polymersomes. The amount of polymersomes per cell nuclei was determined using FIJI (*N* > 150 cells per condition). Error bars represent the standard deviation.

A short in vitro essay was set up to compare uptake rates of macrophages and tumor cells. Polymersomes were incubated for a certain amount of time with either MDA-MB-231 tumor cells or J774 BALB/c mouse macrophage cells. In Figure 4, the uptake rates are shown for the two individual cell lines. It is clear that macrophages not only take up the nanocarriers more quickly than the tumor cells do, but they also take up more of the polymersomes within the first 120 min. With the macrophages being present in large organs like the liver and spleen with a high blood throughput, injected polymersomes will be efficiently filtered from the blood. Removal of the macrophages would thus be expected to significantly increase circulation time in vivo.

#### 3.2.2. Animal Experiments

**Figure 5:**
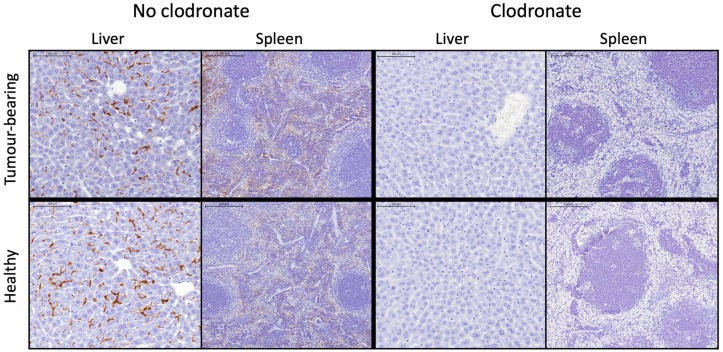
Macrophage staining of the liver and spleen of either healthy or tumor-bearing female Balb/c nude mice, either with or without clodronate liposomes injected 5 days and 1 day before intravenous polymersome injection. The scale bar represents 100 µm for the liver images and 200 µm for the spleen images.

The mice used in these studies were BALB/cAnNRj-Foxn1^nu/nu^ nude mice. These animals are known for their immunodeficiency, which makes it easy to grow human tumors. However, despite their immunodeficiency, they do still have some macrophages, and there is a marked difference in clearance rate of the polymersomes in tumor-bearing versus non-tumor-bearing mice. To determine whether the rapid polymersome clearance was mediated by macrophages, clodronate liposomes were injected in the animals 5 days and 1 day prior to polymersome injection. Clodronate liposomes are known for their toxicity to macrophages, and the administration schedule should ensure complete macrophage ablation at time of polymersome injection [38]. To validate whether the macrophages were indeed destroyed by the clodronate liposomes, both the spleen and the liver were stained for the F4/80 antigen to study the presence of macrophages. In Figure 5, representative tissue sections of liver and spleen tissues of the four different treatment groups can be observed. While the stains indicate F4/80 presence in the control animals (brown), the animals pretreated with clodronate liposomes clearly do not contain any macrophages in their liver and spleen.

**Figure 6:**
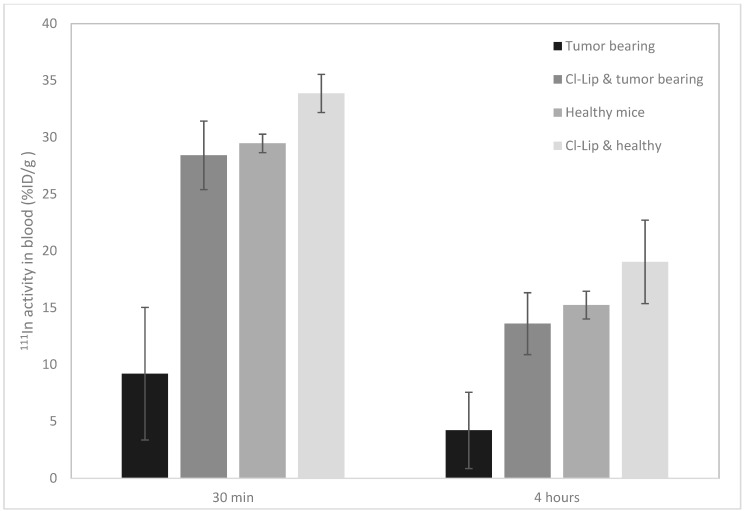
Concentration of ^111^In-polymersomes measured in the blood at 30 min and 4 h p.i. Two of the four treatment groups (labeled Cl-Lip, one with MDA-MB-231 tumors and one without tumors) were injected with clodronate liposomes to deplete their macrophages at 5 days and 1 day before the polymersome injection. The other two groups did not receive clodronate liposome injections, and consisted of one healthy group and one group with MDA-MB-231 tumors.

To determine whether the macrophages were the main cause for the difference in circulation time between healthy and tumor-bearing mice, a blood sample was taken from the animals at 30 min post polymersome injection, and the activity in the blood was again measured at 4 h p.i. upon sacrifice of the animals (Figure 6). The amount of activity in the blood for the four different treatment groups correlates very strongly with the presence or absence of macrophages. The circulation time of polymersomes in tumor-bearing mice which had not undergone the chlodronate liposome treatment was significantly shorter than that in the other treatment groups (*p* < 0.005). There is no significant difference in circulation time between healthy animals, and the healthy and tumor-bearing mice which were pretreated with clodronate liposomes (*t* = 2.2, *d.f.* = 5, *p* = 0.08, and *t* = 1.1, *d.f.* = 4, *p* = 0.3 at 4 h p.i., respectively, with *t* the *t*-value, *d.f.* the degrees of freedom, and *p* the probability). On the other hand, in tumor-bearing mice with macrophages, polymersomes are still rapidly removed from circulation. This implies that tumor presence indeed activates macrophages in the mice, causing the nanoparticles to be removed rapidly from the blood circulation.

**Figure 7:**
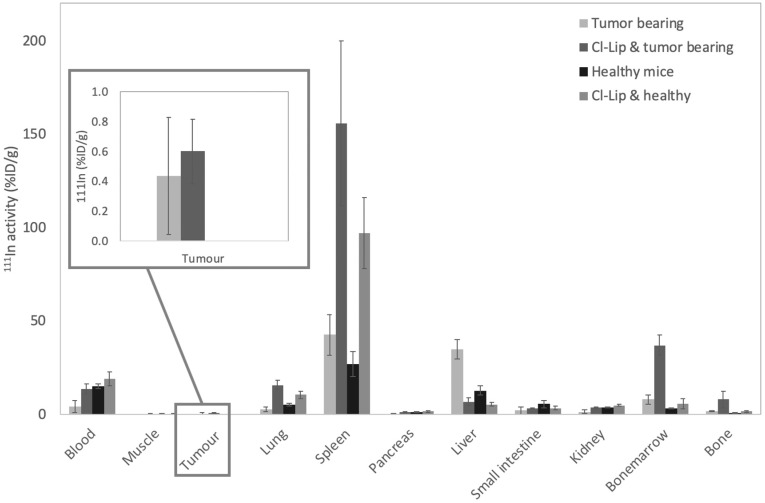
Biodistribution data of 80 nm diameter polymersomes loaded with approximately 1 MBq of ^111^In were injected intravenously in healthy and tumor-bearing Balb/c nude mice. Two of the groups (with and without tumor) were injected with 200 uL of the clodronate liposome solution at 5 days and 1 day prior to polymersome injection. The biodistribution was performed 4 h p.i. Bars represent mean with SD.

Figure 6 shows the biodistribution data at 4 h p.i. for all treatment groups. Next to the activity present in the blood, other significant differences in organ uptake can be found in the lungs, spleen, and liver. While bone marrow uptake appears to be significantly increased in the tumor-bearing mice which received the clodronate liposome treatment, it has to be noted that in these animals, there was hardly any bone marrow present, so the very low bone marrow mass potentially inflated the measured %ID/g value. Pretreatment with clodronate liposomes (and hence absence of macrophages) causes a decrease in liver uptake, and increase in spleen and lung uptake. This appears to be mainly an effect of the clodronate liposome distribution and subsequent macrophage depletion rather than of the polymersomes themselves. While about 80–90% of the body macrophage population are located in the liver [39], even in the control animals whose macrophages were not depleted, polymersomes are preferentially taken up in the spleen (Figure 7). Liver macrophages (Kupffer cells) are eliminated more completely at a much lower clodronate liposome concentration than macrophages in the spleen (0.02 mL vs 0.1 mL per 10 g body weight, respectively) [40]. While several subpopulations of splenic macrophages (the red pulp macrophages (RPM), marginal zone macrophages (MZM), and marginal metallophilic macrophages) are depleted after the clodronate liposome injections [40,41], white pulp macrophages (WPM) and tangible body macrophages in the spleen are affected but not depleted [42]. Nanoparticles have been shown by Demoy et al. to be predominantly captured by the MZM in mice (96% [43]), but slightly more in the WPM than the RPM [44]. The WPM do not express the F4/80 antigen [45], which could explain why, despite the absence of visible macrophages in the F4/80 stained spleens of the mice pretreated with clodronate liposomes (Figure 5), the uptake of the polymersomes in the spleen of these animals is enhanced.

Lung macrophages are not targeted at all through intravenous injection of the clodronate liposomes [40] (they require intratracheal administration), resulting in the increased lung uptake upon depletion of liver and spleen macrophages. Hence, it follows quite logically that when the immune cells of the liver and part of the spleen are depleted, polymersome uptake increases in both the lungs and spleen.

In the end, for efficient tumor targeting, longer circulation half-lives are required than even the 2.3 h in the present study. As can be observed in Figure 7, there was no significant increase in polymersome accumulation at the tumor site for the tumor-bearing mice which had been pretreated with clodronate liposomes. Despite the significantly longer blood circulation time of the polymersomes in the pretreated group, it is still insufficient for therapeutically significant tumor accumulation. Where the circulation half-life of these polymersomes is in the order of two hours, liposomes are generally still in circulation after 24 h, at which they can achieve a tumor accumulation of about 5% ID/g in the MDA-MB-231 tumor model [7,46]. As it takes a longer time for the liposomes to be taken up by the macrophages, their concentration in the blood is initially much larger resulting in the larger fraction to accumulate at the tumor site. Furthermore, it is well established that uptake of nanovesicles via the EPR effect takes time and that it takes several days to reach maximal tumor uptake [47,48]. The lower circulation time of our polymersomes compared to liposomes is likely largely influenced by the degree of PEGylation, as most research indicates that the typical PEG length necessary for nanocarriers to achieve a stealth-like character corresponds to a molecular weight of about 2000, a factor 2 times higher than the PEG length of the block copolymers used in this study [49,50]. Indeed, with a longer PEG, PBd-PEO polymersomes have been found to circulate longer in healthy mice (15.8 h and 28.0 h for 1100 g/mol and 2150 g/mol PEG, respectively) [26]. The minimal PEG length requirement is likely because at lower molecular weights, the PEG is no longer flexible enough to prevent opsonization [34].

#### 3.2.3. Comparison to Other Nanoparticle Systems

This study has focused on the circulation time of polymersomes in mice with one type of tumor. However, the results obtained can be widely applied to a number of nanoparticle systems. Despite only one other study having directly studied the circulation time effect of a tumor presence [22], the results they obtained with their system closely match ours. They considered the influence of three different tumor types, which all demonstrated shorter circulation times. This, together with the two tumor models [21] studied by our group, lead us to believe this is a universal phenomenon. Despite there not being any studies on liposome circulation time directly studying the influence of tumor burden, Gabizon et al. have studied the biodistribution of a number of liposome compositions [51]. While using the same liposomes in their study, they unfortunately switched mouse type from Swiss Webster female mice to BALB/c female mice when going from assessing circulation time in healthy vs tumor-bearing mice, making it difficult to prove the effect of the tumor burden. Their data, however, shows that about 20% ID is present in the blood after 25 h for the healthy mice vs less than 10% ID in the tumor-bearing mice, suggesting that the effect of the tumor presence has a significant effect on circulation time here as well. While more research is definitely required to draw definite conclusions, the tumor influence on circulation time of nanoparticles appears to be a universal phenomenon, at least in mice. However, as it is mainly the spleen which is responsible for filtering the polymersomes from the blood circulation, one has to keep in mind that the results obtained in this study cannot directly be translated to humans. Given that the spleen greatly varies between species, where the spleen of mice is nonsinusoidal, and that of humans (and rats) is sinusoidal, translational studies are essential to determine the extent of this phenomenon [39]. Clearly, though, it is not sufficient to study the circulation behavior of nanoparticles solely in healthy mice. The large discrepancies in circulation time between the healthy and tumor-bearing mice in this study have shown the need for proper assessment of any nanoparticles designed for tumor targeting. A detailed understanding of the tumor activation of the immune system and its subsequent effect on nanoparticle circulation time could very well prove to be essential for the successful translation of any type of nanoparticle system to clinical applications.

## 4. Conclusions

In this work, we have studied the influence of tumor presence on the circulation time of polymeric nanocarriers upon intravenous injection. A large difference in circulation time has been observed between healthy and tumor-bearing mice, with circulation half-lives of 117 min (*R*^2^ = 0.95) and 5 min (*R*^2^ = 0.98), respectively. Cell studies have shown an increased uptake rate of the polymersomes in macrophages vs tumor cell cultures. Through the administration of clodronate liposomes before the intravenous polymersome injection, we have been able to deplete both spleen and liver of macrophages. Subsequent circulation time studies have shown that the removal of macrophages in the liver and spleen allowed the polymersomes in tumor-bearing mice to circulate equally long as in healthy mice, pointing to macrophage activation as the main reason for the observed difference in circulation time. The findings in this study greatly emphasize the need for evaluation of novel nanoparticle therapeutic agents in mice with and without tumor, as the results in healthy animals cannot directly be translated to circulation half-lives in tumor-bearing animals.
